# Recognition of change in the reform of forensic mental health by clinical practitioners: a questionnaire survey in Japan

**DOI:** 10.1186/1744-859X-13-9

**Published:** 2014-03-29

**Authors:** Akihiro Shiina, Masaomi Iyo, Akira Yoshizumi, Naotsugu Hirabayashi

**Affiliations:** 1 Department of Psychiatry, Chiba University Hospital, Chiba 260-0877, Japan; 2 Department of Psychiatry, National Hospital Organization Sakakibara Hospital, Mie 514-1292, Japan; 3 Department of Psychiatry, National Center of Neurology and Psychiatry, Tokyo 187-8551, Japan

**Keywords:** Forensic mental health, Offenders with mental disorders, Medical Treatment and Supervision (MTS) Act, Mental Health and Welfare (MHW) Law, Questionnaire survey, Community mental health

## Abstract

In Japan, new legislation regarding forensic mental health, namely, the Act on Medical Care and Treatment for Persons Who Have Caused Serious Cases under the Condition of Insanity (Medical Treatment and Supervision Act (MTS Act)) was enforced in 2005, although community mental health care remains largely unchanged. We surveyed local clinical psychiatrists by questionnaire to gather information on the influence of the MTS Act on clinical mental health practice. We sent a paper questionnaire to almost all the psychiatrists in the Chiba prefecture, 56% of whom (*N* = 306) responded. The participants felt that the MTS Act had minimal direct impact on community mental health care. However, some relatively new schemes such as a multiple disciplinary team approach or supervised outpatient care are given more attention than before. These results suggest that this new forensic mental health legislation may assist in the spread of new paradigms into clinical practice.

## Introduction

The need for sophisticated forensic mental health practices has increased, as global trends move toward the deinstitutionalization of patients with mental disorders [1]. It is clear that many countries have their own forensic mental health systems which link together different disciplines [2].

For many years, Japan had no specific legal provision for offenders with mental disorders [3]. Such offenders were treated under the Mental Health and Welfare (MHW) Law. According to this legislation, patients with mental disorders who were potentially dangerous and capable of harming themselves or others were hospitalized under a prefectural government order. This system of official involuntary hospitalization (OIH) is completely independent of the criminal justice system [4]. This led some lawyers to claim that the human rights of these patients were not being properly assured. Meanwhile, some psychiatrists suggested the need for special hospitals with a secure structure and adequate staff to provide appropriate care for offenders with mental disorders [5].

To address these problems, the Japanese forensic mental health system was reformed at a time that coincided with the enforcement of the Act on Medical Care and Treatment for the Persons Who Had Caused Serious Cases under the Condition of Insanity, or the Medical Treatment and Supervision (MTS) Act, in 2005 [6]. Under this new scheme, individuals committing a serious criminal offense in a state of insanity or diminished responsibility would be dealt with in a judicial administrative framework. The court would order offenders to undergo specialized medical services at an appropriate hospital. In addition, offenders would be supervised by a rehabilitation coordinator who was part of the probation service. Since the MTS Act was enforced some 8 years ago, several papers have been published reporting on the outcome of this new system [7,8].

At the same time, the conventional scheme of OIH under the MHW Law remains almost unchanged. Currently, offenders diagnosed with a mental disorder who commit less serious crimes while under the influence of their disorder and who require immediate medical care are subjected to OIH. In contrast to the MTS Act, Japan has no system in place to monitor patients who are hospitalized under the MHW Law. Although a few surveys have been performed [9], the outcome of these patients has not been adequately examined.

Generally speaking, reformation of the mental health care system results in significant changes of the immediate environment of patients with mental disorders [10-12]. However, it is doubtful whether enforcement of the MTS Act will dramatically change mental health care practice in Japan. According to official reports, the number of subjects for whom the MTS Act is applicable may be as low as 400 individuals per year [13]. This number is perhaps too small to influence the general functioning of the entire mental health care system in Japan. In addition, most general psychiatrists do not engage with the MTS Act. In a previous study, we ascertained that the attitude of clinical psychiatrists toward forensic mental health had not changed since the MTS Act came into force [14].

Nonetheless, it is possible that clinical psychiatrists have felt some indirect effects in everyday practice after enforcement of the MTS Act, such as a change of the characteristics of patients, the development of multiple disciplinary teams (MDT) or collaborations with related facilities, and the worsening of discrimination against patients, factors which were prevalent before the MTS Act was implemented [15,16]. It is more likely that the development of forensic mental health systems will be beneficial to clinical psychiatry if the enforced changes are not limited to the forensic setting, but applicable to general clinical practice. Conversely, we should be vigilant of any adverse effects of the MTS Act on general psychiatry. To examine these issues, we have surveyed local psychiatrists by questionnaire to gather opinions on the practice of clinical mental health.

## Materials and methods

To achieve our aim, we attempted to canvass the opinions of all psychiatrists working in the Chiba prefecture. We created a paper questionnaire to gather the views of participants. Using official databases, we created a list of all facilities in the Chiba prefecture equipped with a psychiatric outpatients unit, including all clinics and hospitals. Our questionnaire was sent to all the facilities on our list, with a request that the answer sheet be returned to us. The data examined included the demographics of the participants, their knowledge of changes in mental health practice and the environment around inpatient care, and their opinion of the mental health care system (Table 1).

**Table 1:** Contents of the questionnaire

**Head**	**Content**
Characteristics of the participants	Age, sex, years of experience as a medical practitioner, with/without a designated physician’s license for the MHW law, experience in assessing official involuntary hospitalizations (OIH), with/without a judgment physician’s license for the MTS Act, and experience of the MTS Act as a mental health reviewer
Changes in mental health practices from 2005 to 2010 (this series of questions is applicable only for participants engaged in clinical mental health care for more than 5 years)	Increased paperwork, greater curiosity about multiple disciplinary teams, increased workload, widespread knowledge of mental health care, greater concern about the mental health system, increase in patients with mild symptoms, development of mental health care, more frequent talks about human rights, ease of collaboration with other facilities, increased opportunity to treat patients with severe symptoms, and increased discrimination against patients with mental disorders
The recognition of changes within inpatient care settings from 2005 to 2010 (this series of question is applicable only for designated physicians working at hospitals which accept cases of OIH)	Clinical severity of OIH patients, difficulty in discharging OIH patients, recurrent hospitalization of OIH patients, and violence in hospitals
Optimization of mental health care practices	Sharing the task of assessing for OIH, follow-up for patients who do not meet the threshold for OIH, assessing the discharge of patients in OIH, and supervision of patients who undergo repeated OIH

This survey was conducted from November 2011 to February 2012. The collected data were analyzed using PASW Statistic 18®.

### Ethical issues

This survey focused on the opinions of clinical practitioners and thus did not gather personal information on any patient. We registered this survey on the Clinical Trials Registry (CTR) of the University Hospital Medical Information Network (UMIN, Tokyo, Japan), with the unique trial number UMIN000006824.

This research was supported by a grant to Naotsugu Hirabayashi from the Ministry of Health, Labour and Welfare in Japan, as part of a research project entitled ‘Research on the appropriate care and improvement of reintegration into society of patients with mental disorders who have committed serious offences’. A portion of the results were collected as research for working papers, according to the demands of the Ministry of Health, Labour and Welfare and were sent to the government and associated colleagues.

## Results

### Response rate

We sent the questionnaires to 181 facilities in the Chiba prefecture and received responses from 79 facilities (43.6%), of which 20 were mental hospitals, 9 were general hospitals with psychiatric wards, 7 were general hospitals without psychiatric wards, 41 were mental clinics, and 2 were unclassified facilities. A total of 306 answer sheets were returned. The number of respondents accounted for 57.6% of all psychiatrists working in the Chiba prefecture (*n* = 531) [17].

### The characteristics of the participants

The mean age of the respondents was 47.0 ± 13.5 (mean ± standard deviation). Of the respondents, 243 were male, 60 were female, and 3 did not document. The mean term of experience as a clinical practitioner was 18.8 years. Two hundred and thirty-three (approximately three quarters) of the respondents held a designated physician’s license for the MHW Law. Of these, 49 had no experience of assessment for OIH, while 18 conducted assessments for OIH more than once in a month. Thirty-nine (approximately one tenth) respondents held a judgment physician’s license for the MTS Act. Three of these respondents had no professional experience of the MTS Act as a mental health reviewer, while 3 had used it in excess of ten times.

### Changes in mental health practices from 2005 to 2010

**Figure 1:**
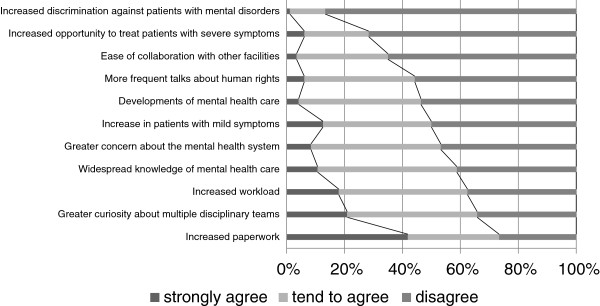
**Changes in mental health practices from 2005 to 2010.** This figure shows the distribution of opinions for each listed topic. A total of 260–263 responses were obtained from psychiatrists engaged in clinical mental health care for more than 5 years.

We asked the participants to answer questions about changes they had noted in mental health practice in the period 2005 to 2010, using a rating scale of ‘strongly agree’, ‘tend to agree’, and ‘disagree’. This series of questions was only applicable for participants practicing in clinical mental health for more than 5 years. A total of 260–263 valid answers were obtained. The response ‘strongly agree’ was most frequently elicited in relation to ‘Increased paperwork tasks’. A total of 41.8% of the participants chose this option. This was followed by ‘Greater curiosity about multiple MDT’, as highlighted by 20.9% of respondents. In contrast, 86.6% of the participants disagreed with the statement ‘Increased discrimination against patients with mental disorders’ (Figure 1).

**Figure 2:**
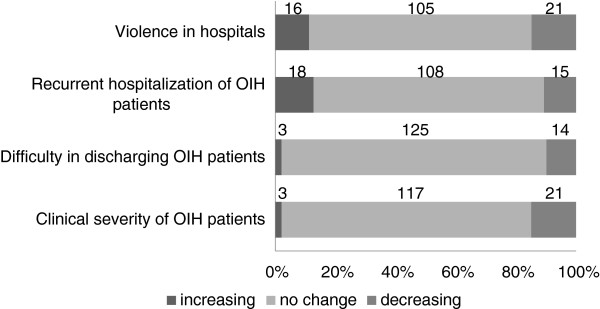
**Changes in the inpatient care setting from 2005 to 2010.** This figure shows the distribution of opinions for each listed topic. A total of 141–142 responses were obtained from designated physicians working at mental hospitals accepting cases of official involuntary hospitalization (OIH).

We also canvassed views on changes in inpatient care settings, in the period 2005 to 2010. This series of questions was applicable only to designated physicians working at hospitals accepting cases of OIH. Between 141 and 142 valid answers were returned. The majority of the participants replied ‘no change’ to questions relating to ‘Clinical severity of OIH patients’, ‘Difficulty in discharging OIH patients’, ‘Recurrent hospitalization of OIH patients’, and ‘Violence in hospitals’ (Figure 2).

### Optimization of mental health care practices

Finally, we asked all participants for their opinions on the mental health system in Japan. This section consisted of four questions with multiple choice responses.

In relation to sharing the task of assessing patients under OIH, 104 (39.3%) participants replied that multiple psychiatrists should be engaged in this role. Fewer participants, i.e., 33 (12.1%), thought that this work should be limited to designated psychiatrists with appropriate skills. For the treatment of individuals who did not meet the threshold for OIH, 144 participants (52.4%) felt that there was a necessity for a new scheme providing official follow-up. Only eight participants (2.9%) opposed official follow-up. Considering the assessments for discharge of patients in OIH, 149 participants (54.4%) thought the current scheme functional. However, 73 participants (26.6%) advocated a stricter process of assessment such as introducing a new panel. In relation to the supervision of patients repeatedly assessed for OIH, 157 participants (57.7%) supported the introduction of a new scheme that forces these patients to make periodic visits to a mental clinic.

### Cross tabulation

Cross tabulation analysis revealed that participants working in hospitals which accept OIH cases tended not to believe that there was an increase in patients with mild symptoms, compared with those working in other types of facilities (chi-square test: *df* = 2, chi-square = 10.48, *P* = 0.0053).

## Discussion

We conducted a questionnaire survey to collect the opinions of mental health practitioners about the changes in the current practice of mental health care and preferred possible future changes. We received answers from more than half of the psychiatrists in the Chiba prefecture and thus believe that these results may be representative of mental health care providers in the Chiba prefecture.

The task of assessing for OIH seems to fall to a limited number of psychiatrists who are actively engaged in mental health practice. In our survey, many see this as problematic, as it appears to create an unfair burden on a small pool of psychiatrists. On the other hand, some participants felt that only adequately trained psychiatrists should deal with legal cases such as those requiring OIH. Our results indicate a need to both increase the number of practitioners capable of performing these assessments while also improving the skill levels and confidence of the psychiatrists carrying out this specific task. This type of training structure is already in place to support practitioners working with the MTS Act [18].

Many psychiatrists feel that their daily workload, particularly their paperwork, has increased. Although overall opinion suggested that the symptoms of patients were becoming less severe, this was not supported by practitioners at facilities which accepted OIH patients. It seems plausible that patients with milder symptoms are now more likely to visit a mental clinic than previously, and not that symptom severity has decreased over the patient population as a whole.

In practice, a significant number of participants tended to consider MDT in their routine work. Recently, several workshops focusing on MDT have been held [19,20]. It is likely that the MTS Act which emphasizes importance of the MDT has resulted in a greater uptake of MDT consultations in routine practice.

Generally, the participants did not feel any appreciable change in care delivery around OIH. The worry that the MTS Act would lead to increased discrimination against patients with mental disorders has been unfounded, at least amongst practitioners. Many participants felt that patients who repeatedly performed antisocial acts did require special measures. Under the MTS Act, patients undergo mandatory supervision by a rehabilitation coordinator. Some practitioners would welcome the introduction of a similar scheme in the community mental health setting.

## Conclusion

We performed a questionnaire survey of community mental health practitioners to obtain their opinions on changes in clinical practice after enforcement of the MTS Act. Most participants felt that the MTS Act had no appreciable direct impact on community mental health care. However, relatively new schemes, such as MDT practice or supervised outpatients care are gaining greater attention. The MTS Act might contribute to the spread of these practices.

## Competing interests

The authors declare that they have no competing interests.

## Authors’ contributions

AS, MI, and AY designed the questionnaire. AS carried out this survey. AS and MI wrote the manuscript. NH was the administrator of the whole the study. All authors read and approved the final manuscript.
